# Nivolumab-induced IgA nephropathy in a patient with advanced gastric cancer: A case report

**DOI:** 10.1097/MD.0000000000020464

**Published:** 2020-05-22

**Authors:** Katsuyuki Tanabe, Hiromitsu Kanzaki, Takahira Wada, Yuri Nakashima, Hitoshi Sugiyama, Hiroyuki Okada, Jun Wada

**Affiliations:** a Department of Nephrology, Rheumatology, Endocrinology and Metabolism; b Department of Gastroenterology and Hepatology; c Department of Human Resource Development of Dialysis Therapy for Kidney Disease, Okayama University Graduate School of Medicine, Dentistry and Pharmaceutical Sciences, Okayama, Japan.

**Keywords:** case report, gastric cancer, IgA nephropathy, nivolumab, steroid

## Abstract

**Introduction:**

Immune checkpoint inhibitors including nivolumab, an antibody against programmed death-1, have been increasingly introduced in various cancer treatment regimens, and are reported to be associated with immune-related adverse events. Nivolumab-induced renal injury is generally caused by acute interstitial nephritis and is managed by drug discontinuation and steroid therapy. Although this agent can infrequently induce glomerulonephritis, the pathogenesis and therapeutic strategy remain undetermined.

**Patient concerns:**

A 78-year-old man was diagnosed with advanced gastric cancer with portal thrombosis. First- and second-line chemotherapies were ineffective; thus, nivolumab monotherapy was initiated. Although it effectively prevented tumor growth, proteinuria and microhematuria appeared 2 months later. Despite drug discontinuation, serum creatinine progressively increased from 0.72 to 1.45 mg/dL. Renal biopsy revealed mesangial IgA and C3 deposition in immunofluorescence analysis and mesangial proliferation with crescent formation in light microscopy.

**Diagnosis:**

The patient was diagnosed with IgA nephropathy. Based on the temporal relationship between the nivolumab therapy and abnormal urinalysis, IgA nephropathy was considered to have been induced by nivolumab.

**Interventions:**

A moderate dose (0.6 mg/kg/day) of prednisolone was orally administrated, with tapering biweekly.

**Outcomes:**

Steroid therapy stabilized his serum creatinine levels and markedly reduced proteinuria. However, bacterial pneumonia substantially impaired his performance status; thus, nivolumab could not be restarted despite tumor regrowth.

**Lessons:**

IgA nephropathy should be recognized as an uncommon renal adverse event during nivolumab therapy. After drug discontinuation, nivolumab-induced IgA nephropathy is likely to respond to moderate doses of steroid therapy with early tapering. However, more evidence is needed to determine whether nivolumab can be safely restarted during or after steroid therapy.

## 1 Introduction

Traditional chemotherapy using cytotoxic agents, such as cisplatin, has been associated with renal injury.^[1]^ In most cases, renal histology is consistent with acute tubular necrosis (ATN). As drug discontinuation is the only therapeutic approach to chemotherapy-associated ATN, renal injury greatly limits cancer treatment.^[2]^ In the past decade, a variety of molecular targeting drugs were introduced into clinical practice. Some of these drugs, such as vascular endothelial growth factor receptor or epidermal growth factor receptor blocking agents, can induce glomerular injury rather than ATN.^[3]^ Histological evaluation is increasingly important to clarify the pathogenesis of chemotherapy-associated renal injury.

Nivolumab is a fully human immunoglobulin G4 (IgG4) antibody directed against programmed death -1 (PD-1). PD-1 is a negative regulatory receptor expressed on the surface of activated T cells and B cells; thus it acts as an immune checkpoint.^[4]^ Inhibitory effects of nivolumab on immune checkpoints enhance the antineoplastic immune response.^[5]^ Cytotoxic T-lymphocyte-associated protein-4 (CTLA-4) is another immune checkpoint; additionally, human anti-CTLA-4 IgG1 antibody, ipilimumab, has antineoplastic activity.^[5]^ These immune checkpoint inhibitors have been incorporated into many cancer treatment regimens. However, owing to their ability to enhance immune responses, anticancer therapies with immune checkpoint inhibitors are sometimes associated with various immune-related adverse events (irAEs), including thyroid disorders, type 1 diabetes mellitus, colitis, encephalitis, and interstitial pneumonitis.^[6]^

Although the kidney is infrequently involved in immune checkpoint inhibitor-induced irAEs, nivolumab can cause acute kidney injury due to acute interstitial nephritis in most cases with renal events.^[7]^ Such acute kidney injury events can be managed by drug discontinuation and/or steroid therapy. However, according to some recent case reports, nivolumab may be associated with glomerular disorders, including nephrotic syndrome and glomerulonephritis.^[8]^ Unfortunately, nivolumab-induced glomerular disorders and their therapeutic strategies have not been well characterized, compared to acute interstitial nephritis, due to insufficient data. In this case report, we present a patient who was diagnosed with IgA nephropathy after nivolumab therapy against advanced gastric cancer and discuss the pathogenesis and potential therapeutic strategy. Written informed consent was provided by the patient for publication of this case report.

## 2 Case presentation

**Figure 1:**
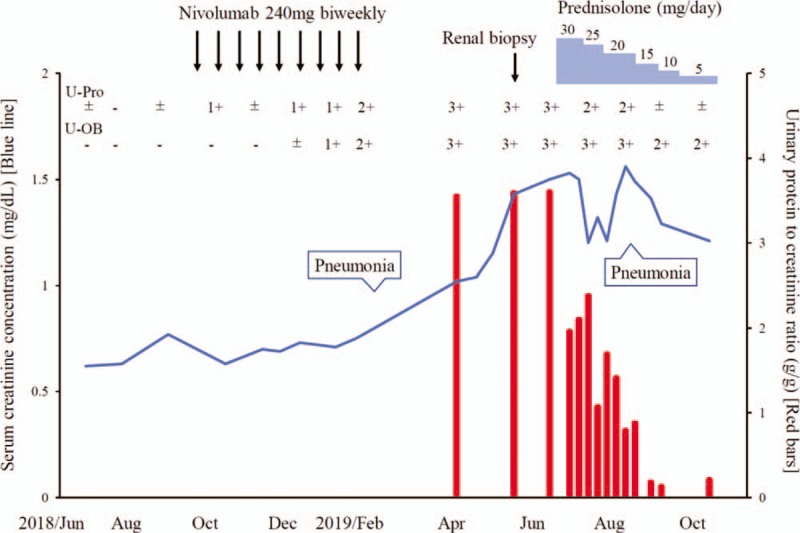
Clinical course and treatments of the patient. U-Pro = urinary protein, U-OB = urinary occult blood.

A 78-year-old Japanese man with type 2 diabetes mellitus was diagnosed with advanced gastric cancer and portal vein tumor thrombus (T3, N3, M1; stage IV) in August 2017. His hemoglobin A1c level was well controlled in the range of 5.7% to 6.0% by the administration of 5 mg/day of linagliptin. First-line (S-1 + oxaliplatin) and second-line (ramucirumab + paclitaxel) chemotherapies were discontinued owing to disease progression, and nivolumab monotherapy (240 mg, biweekly) was started as third-line therapy in September 2018. Until then, urinalysis revealed only trace proteinuria and his serum creatinine concentration was between 0.64 and 0.72 mg/dL. Nivolumab therapy effectively prevented the growth of the primary gastric tumor and normalized the elevated tumor marker; the serum level of carcinoembryonic antigen decreased from 41.8 ng/mL to 4.9 ng/mL. However, 2 months later, urinary protein 2+ and occult blood 2+ were noted in dipstick tests. Nivolumab administration was discontinued owing to the development of bacterial pneumonia in February 2019; the drug was decided to be withheld until his performance status improved. Nevertheless, the patient presented with massive proteinuria (3+ on dipstick; urinary protein to creatinine ratio, 3.59 g/g of creatinine) and hematuria (>100/high power field) and showed an increased serum creatinine concentration up to 1.45 mg/dL in May 2019 (Fig. 1).

**Figure 2:**
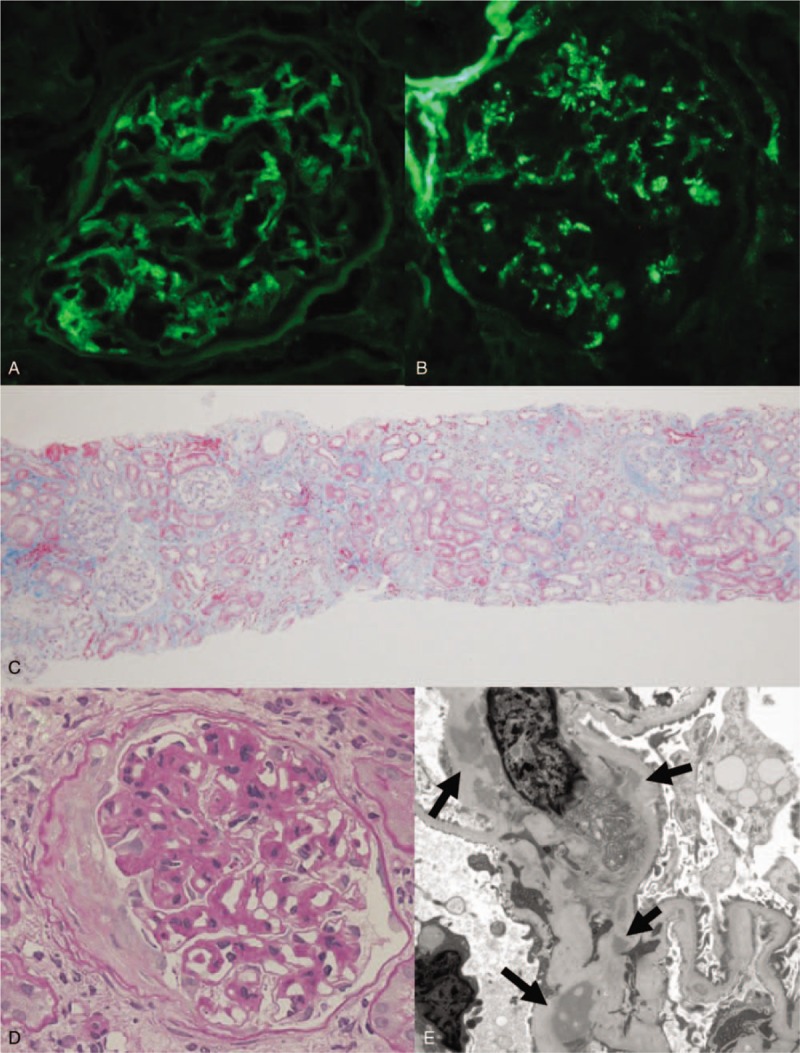
(A, B) Immunofluorescence image for IgA and C3 deposition, respectively (original magnification ×400). (C) Masson-trichrome staining showing tubular atrophy and focal inflammatory infiltration (original magnification ×100). (D) Periodic acid-Schiff staining of glomerulus with mesangial proliferation and fibro-cellular crescent (original magnification ×400). (E) Electron microscopic image showing paramesangial electron dense deposits (arrows, original magnification ×8000).

At the time of nephrology consultation, his blood pressure was 132/70 mmHg and heart rate was 88 beats/min; he had mild pitting edema and no purpura in the lower limbs. In addition to renal dysfunction and proteinuria, as mentioned above, anemia associated with gastric cancer was observed; moreover, the liver function test and electrolyte panel were within the normal range. In the immunological evaluation, serum IgA was increased to 538.8 mg/dL, complement levels were normal (IgA/C3 ratio was 6.24), and cryoglobulin, anti-nuclear antibody, and both myeloperoxidase- and proteinase 3-anti-neutrophil cytoplasmic antigen (ANCA) were negative. Renal morphology on abdominal computed tomography was normal. Renal biopsy was performed for histological evaluation. Immunofluorescence revealed IgA and C3 depositions in the mesangial area (Fig. 2A and B, respectively). Light microscopy revealed moderate tubular atrophy and focal interstitial inflammatory infiltration on Masson trichrome staining (Fig. 2C) and mesangial proliferation with fibro-cellular crescent formation on Periodic-Schiff staining (Fig. 2D). Electron microscopy revealed dense deposits in the paramesangial area (Fig. 2E; indicated by arrows). Based on these histological findings, the patient was diagnosed with IgA nephropathy (MSET-C score; M1, S0, E0, T1, C1). Based on the temporal relationship between the nivolumab therapy and abnormal urinalysis, IgA nephropathy was considered to have been induced by nivolumab.

During the discontinuation of nivolumab, tumor regrowth was observed. However, retreatment with nivolumab was limited by the progressive renal injury. To prevent the deterioration of renal function, a moderate dose of prednisolone (0.6 mg/kg of body weight/day) was orally administered from June 2019. Steroid therapy with tapering biweekly stabilized his serum creatinine concentration between 1.2 and 1.4 mg/dL and markedly decreased his proteinuria to 0.18 g/g of creatinine (Fig. 1). The initial increase in blood glucose needed temporary insulin therapy, but hyperglycemia was promptly improved with steroid tapering and could be managed using original diabetic medication. Although nivolumab re-administration was considered to prevent tumor regrowth, bacterial pneumonia recurrence substantially impaired his performance status; thus, it was difficult to reinitiate nivolumab therapy despite the stabilization of his renal function.

## 3 Discussion

Immune checkpoint inhibitors, including nivolumab, have been increasingly associated with various irAEs in cancer patients, with the expansion of their indications. Notably, some clinical studies have shown that the development of irAEs is associated with better prognosis,^[9,10]^ suggesting the importance of irAE management during immune checkpoint inhibitor therapy. The incidence of renal involvement related to nivolumab monotherapy was estimated to be approximately 2%.^[11]^ However, a recent report suggests that low-grade renal involvements may be underestimated in nivolumab therapy.^[7]^ In contrast to other irAEs such as thyroid disorder and diabetes mellitus, there has been no evidence that nivolumab-induced renal adverse events can be associated with better patient survival. Nevertheless, it is critical, for the continuation of cancer chemotherapy, that the progression of renal injury is effectively managed.

The most commonly reported renal involvements in nivolumab therapy is acute interstitial nephritis.^[7]^ In this process, nivolumab is postulated to induce direct lymphocyte infiltration into the renal interstitium or enhance the potential interstitial nephritis due to other medications.^[12,13]^ Recent case reports and case series have suggested that nivolumab may be associated with glomerulonephritis. According to a recent literature review,^[8]^ nivolumab monotherapy or combined therapy with other immune checkpoint inhibitors can induce ANCA-positive or ANCA-negative necrotizing glomerulonephritis. Furthermore, 2 cases of biopsy-proven IgA nephropathy associated with nivolumab therapy as monotherapy^[14]^ or in combination with ipilimumab^[8]^ have been reported. Based on many basic studies, primary IgA nephropathy is considered to be caused by the production of underglycosylated IgA1 and autoantibodies directed against such abnormal IgA1 molecules and subsequent immune complex formation and mesangial deposition, leading to glomerular inflammation.^[15]^ Therefore, immune disturbance should play a pivotal role in the pathogenesis of primary IgA nephropathy. Although mechanisms underlying nivolumab-induced renal injury remain undetermined, given its higher prevalence in the Asian population,^[16]^ nivolumab therapy may unmask subclinical IgA nephropathy by enhancing immune responses.

Therapeutic strategies against nivolumab-induced acute interstitial nephritis have been established and depend on its severity. Moderate renal injury defined as increased serum creatinine to two- to three-fold above baseline can be managed by moderate doses of prednisone (0.5–1 mg/kg/day) and withholding nivolumab therapy, whereas severe renal injury with a greater increase in serum creatinine level should be initially treated with higher doses of prednisone or methylprednisolone (1–2 mg/kg/day) and withdrawal of nivolumab.^[17]^ The most appropriate strategy by which nivolumab-induced glomerulonephritis can be managed remains unclear as there is not much data available in this regard. Some cases of nivolumab-induced glomerulonephritis were treated with drug discontinuation and high doses of prednisone alone or in combination with other immunosuppressants, leading to complete or partial renal recovery.^[8]^ Although there is still controversy on whether moderate to high doses of steroids can promote tumor growth and poor outcomes in patients treated with nivolumab, obvious deleterious effects of steroid therapy on the clinical effectiveness of nivolumab in cancer patients have not been confirmed to date.^[18]^ In the previous two reports on nivolumab-induced IgA nephropathy mentioned above, the first case with nivolumab monotherapy was followed by only drug discontinuation.^[14]^ The patient's proteinuria was markedly improved and the increased serum creatinine was stabilized; this clinical course appears to be opposed to that of the present case. In the second case of nivolumab therapy combined with ipilimumab, 0.5 mg/kg of body weight/day of prednisone was administrated in addition to drug discontinuation.^[8]^ Such treatment was, at least transiently, effective in achieving remission of glomerulonephritis. Notably, tumor growth during follow-up after discontinuation of nivolumab was not observed in either case.^[8,14]^ In contrast, the present case showed both progressive renal injury and gastric tumor growth during nivolumab withdrawal. Despite improvement of glomerulonephritis by steroid therapy, nivolumab re-administration was withheld due to impaired performance status. As most cases of nivolumab-induced glomerulonephritis to date required drug discontinuation regardless of prednisone treatment,^[8]^ whether nivolumab can be safely re-administrated after or during steroid therapy without relapse of glomerulonephritis remains unclear.

## 4 Conclusion

In conclusion, it should be recognized that IgA nephropathy may be an uncommon irAE associated with nivolumab therapy and progressive glomerular injury limits the continuation of cancer treatment. This irAE can be controlled by nivolumab discontinuation and moderate doses of steroid therapy with early tapering. However, whether nivolumab therapy can be safely restarted remains unclear. Case reports of nivolumab-induced glomerulonephritis, including IgA nephropathy, must be accumulated to determine the appropriate therapeutic strategies.

## Author contributions

**Conceptualization:** Katsuyuki Tanabe, Hiromitsu Kanzaki.

**Data curation:** Katsuyuki Tanabe, Hiromitsu Kanzaki, Takahira Wada, Yuri Nakashima.

**Project administration:** Jun Wada.

**Resources:** Katsuyuki Tanabe, Hiromitsu Kanzaki, Takahira Wada, Yuri Nakashima.

**Supervision:** Hitoshi Sugiyama, Hiroyuki Okada, Jun Wada.

**Validation:** Katsuyuki Tanabe.

**Visualization:** Takahira Wada.

**Writing – original draft:** Katsuyuki Tanabe.
